# Potassium application methods and natural extracts influence yield components and biochemical traits in Faba bean genotypes

**DOI:** 10.1186/s12870-025-07352-6

**Published:** 2025-09-22

**Authors:** Mohamed Ebaid, Ayman Abdeldaiem Mohamed, Dalia Elhag, Radwa Hamdy, Ahmed M. Saad, Ahmed M. Abdelghany, Sobhi F. Lamlom

**Affiliations:** 1 Plant Production Department, Arid Lands Cultivation Research Institute (ALCRI), City of Scientific Research and Technological Applications (SRTA-City), New Borg El-Arab City, 21934 Alexandria Egypt; 2 Agronomy Department, Faculty of Agriculture, Kafrelsheikh University, Kafr El-Sheikh, Egypt; 3 Agronomy Department, Faculty of Agriculture, Benha University, 13511 Benha, Egypt; 4 Crop Science Department, Faculty of Agriculture, Damanhour University, Damanhour, 22516 Egypt; 5 Plant Production Department, Faculty of Agriculture Saba Basha, Alexandria University, Alexandria, 21531 Egypt

**Keywords:** Potassium, Natural extracts, Faba bean, Biochemical analysis, Seed yield components

## Abstract

This study investigated the interactive effects of potassium application methods (foliar and soil) and natural extracts (yeast, algae, and molasses) on yield components and biochemical characteristics of three faba bean genotypes (Giza-716, Nubaria-4, and Nubaria-5). Field experiments were conducted over two consecutive growing seasons (2022/2023 and 2023/2024) using a split-plot design with four replicates. Results demonstrated significant effects of both fertilizer treatments and cultivar selection on most measured parameters, except for 100-seed weight. Molasses and foliar potassium applications significantly enhanced phosphorus content, seed yield, and yield components, while yeast and algae extract primarily improved nitrogen content. Among genotypes, Giza-716 consistently exhibited superior performance across most measured traits compared to the controls. The Giza-716/molasses treatment combination proved most effective for enhancing most studied traits across both seasons, with specific exceptions: Giza-716 with foliar potassium application maximized seed number per plant in the second season, while Nobaria5 with yeast extract optimized nitrogen content. For protein content enhancement, all three cultivars responded favorably to either yeast or algae extracts. Our findings suggest that combining potassium application with Giza-716 cultivation offers a promising approach for enhancing biochemical traits, seed yield, and yield components in faba bean production systems. Molasses and foliar potassium application proved particularly effective for most parameters evaluated.

## Introduction

Legume crops represent a cornerstone of sustainable agricultural systems worldwide, serving as vital sources of high-quality protein while simultaneously contributing to soil fertility through biological nitrogen fixation. Among these, the faba bean (*Vicia faba* L.), also known as broad bean or horse bean, stands as one of humanity’s oldest cultivated legumes, with archaeological evidence tracing its cultivation back over 10,000 years [1]. This versatile crop serves multiple functions across different regions: as premium animal feed and silage in European agricultural systems, as a staple food source supporting nutritional security across Asia and Africa, and as a critical component in crop rotation systems that reduce dependence on synthetic nitrogen fertilizers [2]. The nutritional profile of faba beans underscores their agricultural and dietary significance, containing approximately 26.1% protein, 58.3% carbohydrates, and 25.0% dietary fiber. However, faba beans also contain elevated concentrations of lectins (hemagglutinins) compared to other legumes, which can present nutritional challenges if not properly processed [3, 4].This dual nature, high nutritional value, coupled with antinutritional factors, highlights the critical importance of optimized production practices that can enhance beneficial compounds while minimizing potentially problematic constituents.

Potassium (K) plays a multifaceted and indispensable role in plant physiology, serving as a key regulator of photosynthesis, assimilate transport, enzyme activation, and the management of oxidative stress. Research has consistently demonstrated that strategic potassium fertilization can significantly enhance both yield potential and nutritional quality in faba beans [5, 6]. Potassium deficiency during critical early growth stages can severely impair nutrient assimilation and partitioning between root and shoot systems, ultimately constraining yield potential and reducing crop resilience to environmental stresses [5, 7, 8]. Beyond its fundamental metabolic functions, potassium is essential for optimizing nitrogen fixation capacity and protein synthesis in legumes. Potassium deficiencies frequently result in reduced nodulation efficiency, decreased seed protein content, and increased accumulation of antinutritional compounds [9, 10]. The method of potassium delivery, whether through soil application or foliar feeding, can significantly influence nutrient use efficiency and crop response. Foliar potassium applications offer several distinct advantages over conventional soil fertilization approaches, including reduced total input requirements, avoidance of soil compaction issues, enhanced nutrient use efficiency, and greater cost-effectiveness [11–13]. Various potassium formulations, including borate, citrate, and NPK-humate complexes, have demonstrated remarkable effectiveness as foliar treatments, enhancing nutrient uptake efficiency, plant vigor, and ultimately improving crop yields [7, 10]. These specialized K formulations not only boost productivity metrics but also improve seed nutritional quality by reducing antinutritional compounds while simultaneously promoting protein synthesis pathways [14].

The application of natural bio-stimulants has emerged as a promising and sustainable approach to crop enhancement, driven by increasing demand for environmentally friendly agricultural practices and reduced synthetic input dependency. Yeast extract represents one of the most promising bio-stimulants, owing to its exceptionally rich composition of amino acids, vitamins, growth-promoting factors, and essential minerals, including sodium, calcium, iron, potassium, phosphorus, sulfur, magnesium, zinc, and silicon [15–18]. The efficacy of yeast extract is largely attributed to its high cytokinin content, which stimulates critical physiological processes including cell division, protein synthesis, and chlorophyll production [19, 20]. Field research has consistently reported significant improvements in multiple growth parameters following yeast application, including enhanced leaf area development, increased shoot biomass accumulation, elevated concentrations of photosynthetic pigments, and ultimately improved flower formation and seed yield, alongside enhanced seed quality attributes. Optimal application rates, typically ranging from 3 to 6 g/L, have been shown to substantially enhance plant height, branching patterns, leaf area expansion, and nutrient content while increasing carbohydrate accumulation and chlorophyll levels [21–23].

Seaweed and algae extracts constitute another highly effective category of biostimulants, containing diverse arrays of beneficial compounds including phytohormones (auxins, cytokinins, gibberellins), betaines, and comprehensive macro- and micronutrient profiles [24, 25]. Research has demonstrated their capacity to improve multiple aspects of plant development, including stem strengthening, leaf area expansion, and promotion of robust root system architecture [26]. Field studies have reported yield increases of 16–20% with optimal algae application rates (4 mL/L), accompanied by improvements in pod number, seed weight, and overall plant vigor indices [27]. These beneficial effects are primarily attributed to enhanced chlorophyll production, improved photosynthetic efficiency, and optimized physiological processes that support reproductive development [24, 26]. Molasses, a byproduct of sugar processing, has gained attention as a cost-effective biostimulant rich in carbohydrates, organic acids, and mineral nutrients [28]. Its application can stimulate beneficial soil microbial activity, enhance nutrient availability, and provide readily available carbon sources for plant metabolism, though its specific effects on faba bean production systems remain underexplored [28, 29].

Despite the documented individual benefits of potassium fertilization strategies and natural bio stimulants, critical knowledge gaps persist regarding their interactive effects, optimal application methods, and cultivar-specific responses in faba bean production systems. The complex interactions between fertilization methods, bio stimulant types, genetic background, and environmental conditions require systematic investigation to develop evidence-based management recommendations. Furthermore, the relative effectiveness of organic amendments compared to conventional fertilization approaches in achieving both productivity and sustainability goals remains incompletely understood. While individual studies have examined various aspects of faba bean nutrition and bio stimulant applications, comprehensive comparative analyses incorporating multiple treatment modalities across diverse genetic backgrounds and environmental conditions are limited. This study was designed to systematically evaluate the effects of different potassium application methods (foliar versus soil application) and natural bio stimulants (molasses, yeast extract, and algae extract) on faba bean productivity, yield components, and seed quality across multiple growing seasons and genetically distinct cultivars.

Research Hypotheses: Foliar potassium application will demonstrate superior efficacy compared to soil application due to enhanced nutrient uptake efficiency, reduced soil fixation losses, and improved translocation to reproductive tissues. Natural bio stimulants (yeast, algae, molasses) will synergistically enhance both yield components and seed nutritional quality through improved photosynthetic efficiency, enhanced nutrient mobilization, and optimized plant growth regulation. Significant genotype × treatment interactions will be observed, with specific cultivar-treatment combinations optimizing both productivity metrics and biochemical traits, thereby enabling precision agriculture approaches. By addressing these hypotheses through comprehensive field experimentation, this research aims to contribute to critical knowledge gaps in legume-based agroecological intensification strategies while providing practical guidance for sustainable faba bean production systems.

## Materials and methods

### Experiment set up, soil conditions

The present study was conducted during the 2022/23 and 2023/24 winter seasons under Delta soil conditions to evaluate the effects of potassium fertilization (foliar and soil application) and foliar natural extracts (yeast, algae, and molasses) on the yield and quality of three faba bean cultivars at different growth stages. The experiment included six treatments: (1) control (distilled water), (2) foliar potassium (K), (3) soil-applied potassium (K), (4) yeast extract, (5) algae extract, and (6) molasses. Foliar potassium was applied in three equal doses (3 cm³/L) of a solution containing 36% K₂O at 40, 60, and 80 days after sowing (DAS). Soil potassium was applied as potassium sulfate (48% K₂O) at a rate of 120 kg/ha¹, split into two equal doses at 40 and 80 DAS. The yeast extract, algae extract, and molasses treatments were also applied as three foliar sprays at 40, 60, and 80 DAS, irrespective of irrigation schedules. The study utilized three Egyptian faba bean (*Vicia faba* L.) cultivars—Nubaria-4, Nubaria-5, and Giza-716 obtained from the Field Crops Research Institute, Agricultural Research Center, Ministry of Agriculture and Land Reclamation, Egypt. These cultivars are publicly available breeding lines, and no specific permits were required for their use in this study. Before planting, a soil analysis was conducted to assess the physicochemical properties of the experimental site. Surface soil samples (0–30 cm depth) were collected, air-dried, ground, and sieved through a 2-mm mesh sieve to prepare for analysis. The soil underwent mechanical and chemical characterization (Table 1), including texture, pH, organic matter content, and nutrient composition. The preceding crop in both growing seasons (2022/23 and 2023/24) was maize, ensuring consistent field conditions.

The field experiment was conducted using a split-plot design with three replications, where main plots were assigned to the three faba bean cultivars (Nubaria-4, Nubaria-5, and Giza-716) and sub-plots contained the six fertilizer treatments. Each experimental unit (sub-plot) measured 3.5 × 3.5 m (12.25 m²), consisting of 5 rows spaced 70 cm apart, with 20 cm between plants within rows, resulting in a plant density of approximately 30 plants per sub-plot.

### Planting of Faba bean genotypes

Soil analysis was applied on soil samples as follows: collecting surface soil samples from the experimental sites at the depth of (0–30 cm), then the samples were subjected to physical treatment of air drying, grinding, sieving using a 2 mm mesh sieve, and some mechanical therapies, followed by some aspects of chemical analysis; Maize was represents the preceding crop in both agricultural seasons; Table (1) illustrates the mechanical and chemical characteristic of the experimental site soil.

**Table 1:** Mechanical and some chemical properties of the investigated soil sample

Soil characteristics	2022/23 season	2023/24 season
Mechanical analysis:		
Coarse sand %	5.09	5.17
Fine sand %	24.88	24.46
Silt %	20.61	20.29
Clay %	49.42	50.08
Textural class	Clay	Clay
Chemical analysis:		
pH (1:2.5)	7.86	7.49
Ca Co_3_%	3.59	3.40
Organic matter %	1.72	1.69
EC (dSm^−1^)^*^	1.89	1.92
Total N%	0.14	0.16
Total P%	1.33	1.46
Total K%	0.57	0.67

### Cultural practices

Introducing 75 kg P_2_O_5_ ha^−1^ calcium, super phosphate (15.5%) was carried out simultaneously during land preparation; followed by hand drilling of faba bean cultivars in experimental units of 3.5 m long and 60 cm wide 5 ridges; planting timing was adjusted to be performed on 15th Nov. in both seasons 2022/23and 203/24; Concerning N fertilizer application, it was in the form of ammonium nitrate; 33.5% has been adjusted to two equal doses with total rate of 48 kg N ha^−1^ and it has been introduced between planting and the first irrigation in the two seasons. Other agricultural practices were done as recommended in the region and outlined.

### Data recorded

At harvesting, ten faba bean plants were chosen from the central ridge of each experimental plot to calculate the following traits studied.

I-Three key parameters were evaluated to assess treatment effects on faba bean productivity: (1) grain yield, calculated as total seed weight per hectare for each entire sub-plot (kg ha⁻¹); (2) total seed number per plant, counted from sampled plants; and (3) 100-seed weight (g), determined by weighing random seed samples to evaluate seed size uniformity. These measurements collectively provide comprehensive insights into both quantitative (yield potential) and qualitative (seed characteristics) aspects of crop performance under different management practices.

II- Chemical analysis:

Nutrient composition was determined using standardized analytical protocols. For each randomly selected sample, nitrogen content was quantified via the Kjeldahl method, potassium through flame photometry, and phosphorus by spectrophotometry [30]. Seed samples (average 15 g) were homogenized by crushing and milling, then sieved through a 0.5 mm mesh for uniform particle size. Total nitrogen percentage was calculated as described in Sect. 2.2.5, with crude protein content derived by multiplying total N% by the standard conversion factor of 6.25. The carbohydrate content was determined using AOAC (2000) protocols. These analyses provided comprehensive nutritional profiling of the faba bean seeds under different experimental conditions.

### Statistical analysis

Data from both growing seasons (2022/23 and 2023/24) were subjected to homogeneity testing and analyzed using a split-plot design where cultivars served as main plots and fertilizer treatments as sub-plots, with seasons and replications as additional factors. Statistical analysis was performed according to Snedecor and Cochran [31] using RStudio (Version 2023.12.1) with R version 4.3.2. The experimental design analysis was implemented using the “agricolae” package (version 1.3.7) for ANOVA and multiple comparisons via Tukey’s HSD test at α = 0.05 significance level. Principal component analysis (PCA) was conducted using “factoextra” and “FactoMineR” packages to identify main sources of variation and relationships among traits, while clustering heatmaps were generated using “pheatmap” package (version 1.0.12) to visualize treatment performance patterns. Path coefficient analysis was performed to quantify direct and indirect effects of yield components on grain yield, and variance partitioning determined the relative contribution of each component to total yield variance. Data visualization utilized “ggplot2” (version 3.4.4), “tidyverse” (version 2.0.0), and “corrplot” packages, with all figures produced at 300 DPI resolution and minimum 10pt font sizes for publication quality.

## Results

### Faba bean performance varies significantly across cultivars, treatments, and growing seasons

**Fig. 1:**
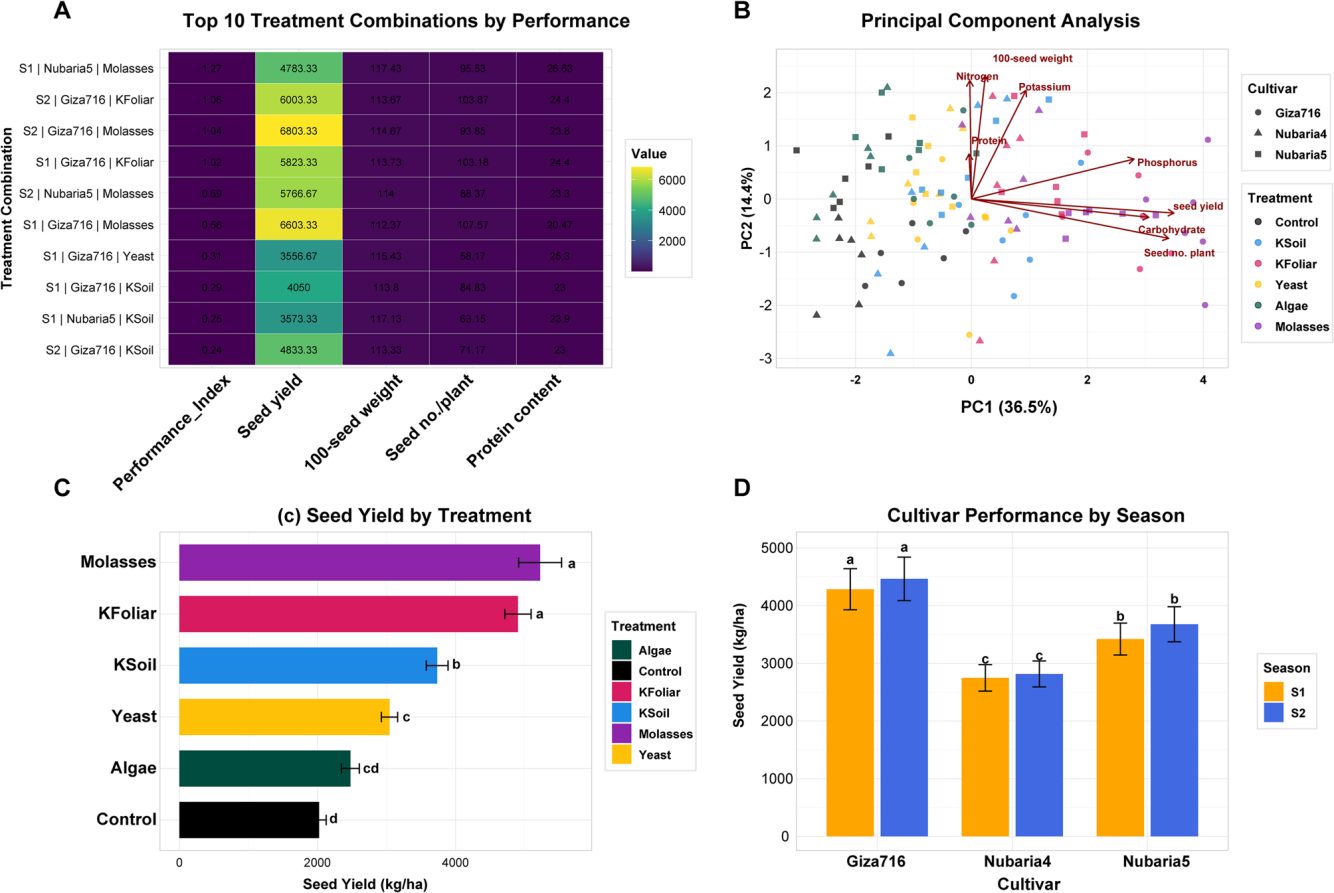
Effects of cultivar, treatment, and growing season on faba bean performance metrics. **A** Top 10 Treatment Combinations by Performance. **B** Principal Component Analysis (PCA) of treatment effects. **C** Seed yield variation among treatment. **D** Seasonal comparison of seed yield between 2022/23 (S1) and 2023/24(S2) growing periods. Error bars represent the standard error of the meaning. Different letters indicate statistically significant differences (*p* < 0.05)

The analysis revealed significant variations in faba bean performance across different cultivars, treatments, and growing seasons **(**Fig. 1**)**. The Yeast treatment in first season (S1) consistently delivered superior results, particularly for Giza-716, achieving the highest Seed yield (SY) (6,803 kg/ha) and excellent hundred seed weight (HSW) (114.67 g). Molasses treatment also showed strong performance, especially for Nubaria-5 in second season (S2), yielding 6,603 kg/ha. In contrast, Control and K-Foliar treatments underperformed, with yields below 4,050 kg/ha. Seasonal effects were pronounced, with S1 generally outperforming S2 across most metrics. Cultivar differences were evident, as Nubaria-4 excelled in S1 while Giza-716 responded best to Yeast treatment. Nutrient parameters (N, P, K) remained stable across treatments, while protein and carbohydrate content showed minimal variation. The Principal Component Analysis clearly distinguished high-performing treatments (Yeast, Algae) from less effective ones (Control, K-Foliar). These findings suggest that optimal faba bean production should combine Yeast treatment with Giza-716 or Nubaria-5 cultivars in S1 growing conditions, while Molasses represents a viable alternative for S2 cultivation. The results underscore the importance of tailored treatment-cultivar-season combinations for maximizing faba bean productivity.

### Agronomic and biochemical traits performance across seasons

**Fig. 2:**
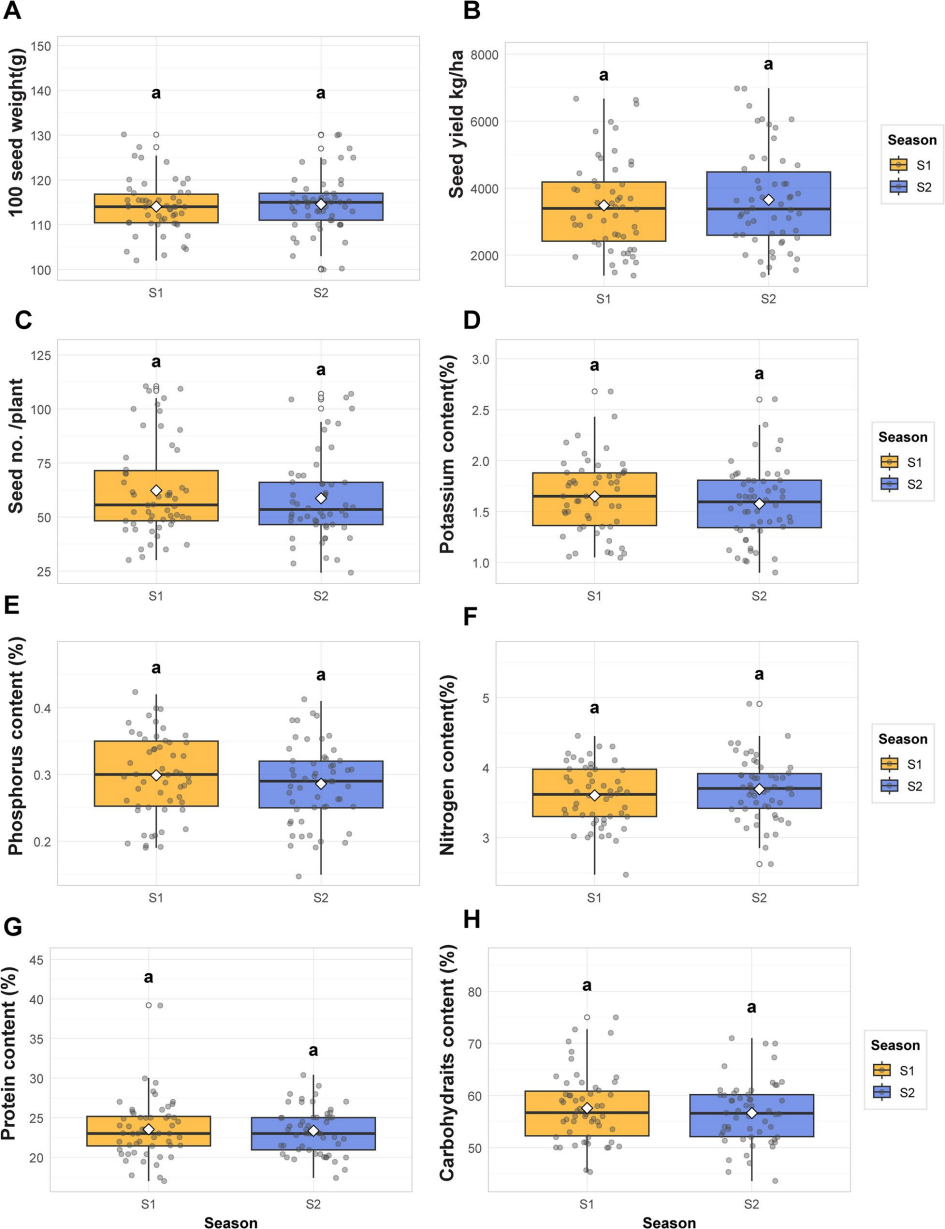
Agronomic and biochemical traits across two seasons **A** hundred seed weight (HSW),** B** seed yield,** C** seed number per pant, **D** potassium content, **E** phosphorus content,** F** nitrogen content, **G** protein content, and **H** carbohydrate content. Letters indicate significant differences (Tukey’s HSD, α = 0.05)

The study examined the influence of two growing seasons (S1 and S2) on various agricultural and biochemical traits, including seed weight, yield, and nutrient content. Significant seasonal effects were observed for several characteristics **(**Fig. 2**)**. SY and HSW were notably higher in one season compared to the other, with S1 demonstrating superior performance in these metrics. Conversely, the number of seeds per plant remained relatively stable across both seasons, indicating minimal seasonal impact on this trait. Nutrient content varied between seasons, with notable differences observed in potassium (K), phosphorus (P), and nitrogen (N) levels. For instance, S1 was associated with higher phosphorus and potassium concentrations, while S2 exhibited elevated nitrogen levels. Protein and carbohydrate content also displayed seasonal variability, with S1 favoring higher protein levels and S2 showing increased carbohydrate content. These results underscore the importance of considering seasonal factors in agricultural planning. For traits like seed weight and yield, S1 appears more favorable, whereas S2 may be preferable for optimizing nitrogen and carbohydrate levels. The stability of seed number across seasons suggests that this trait is less influenced by seasonal changes, making it a more consistent factor in crop production. Overall, the findings highlight the importance of aligning cultivation strategies with seasonal conditions to achieve optimal outcomes.

### Cultivar performance across agronomic and biochemical traits

**Fig. 3:**
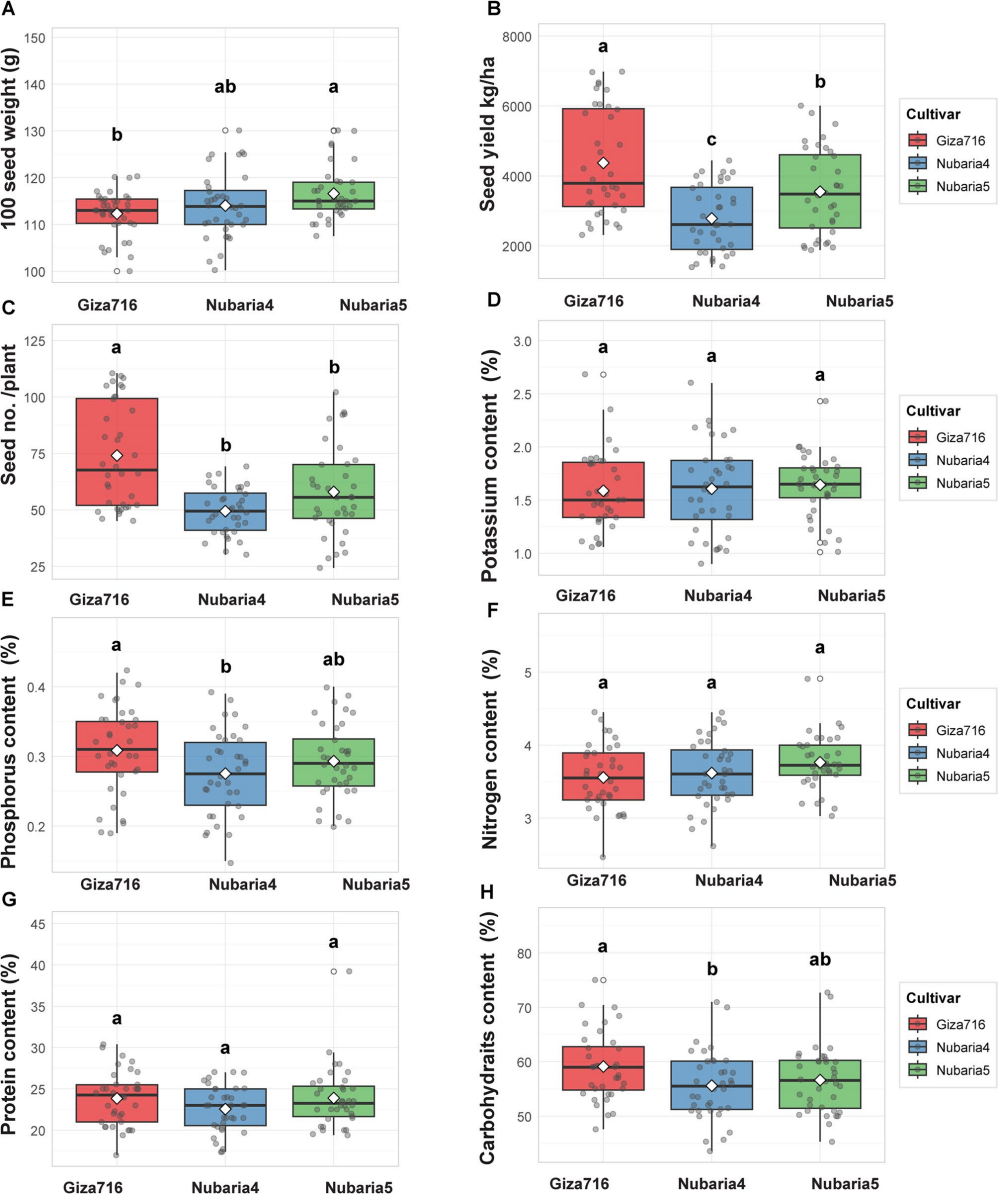
Cultivar performance across agronomic and biochemical traits: **A** 100 seed weight,** B** seed yield,** C** seed number per plant, **D** potassium content,** E** phosphorus content, **F** nitrogen content, **G **protein content, and **H** carbohydrate content. Letters indicate significant differences (Tukey’s HSD, α = 0.05)

Based on the comprehensive statistical analysis presented in the table and systematically illustrated in Fig. 3, detailed cultivar performance patterns reveal distinct genotypic responses across multiple agronomic and biochemical parameters under various treatment regimes.

Giza-716 emerged as the superior cultivar for seed production parameters, achieving the highest SY of 4373 ± 255.9 kg/ha compared to Nubaria-5 (3548 ± 158.6 kg/ha) and Nubaria-4 (2781 ± 203.8 kg/ha), as demonstrated in Fig. 3, where highly significant treatment effects (*p* = 0.001) were observed across both seasons. This cultivar’s higher productivity was further confirmed by its impressive seed number capacity of 74.1 ± 3.8 seeds per plant, which significantly exceeded Nubaria-5 (57.9 ± 1.7) and Nubaria-4 (49.4 ± 3.3). This is illustrated in Fig. 3, where highly significant treatment responses (*p* = 0.001) were observed in both seasonal assessments. The superior performance of Giza-716 was particularly pronounced under optimal treatment conditions, with the Giza-716:Molasses combination achieving remarkable yields of 6703 ± 83.2 kg/ha and Giza-716:K-Foliar interaction producing 103.5 ± 0.89 seeds per plant, representing the highest values recorded across all cultivar-treatment combinations. Conversely, for HSW, Nubaria-5 demonstrated superior performance with 116.6 ± 0.818 g, followed by Nubaria-4 (114.1 ± 1.16 g) and Giza-716 (112.3 ± 0.93 g), as shown in Fig. 3, where non-significant seasonal effects were observed (S1: *p* = 0.18, S2: *p* = 0.21). The optimal HSW performance was achieved by the Nubaria-5:Algae combination at 127.07 ± 1.231 g, while the poorest performance occurred with Giza-716:Control at 103.7 ± 1.28 g, indicating that while Giza-716 excels in seed number and overall yield, Nubaria-5 produces heavier individual seeds.

Regarding nutritional composition, Giza-716 consistently demonstrated superior biochemical characteristics across multiple parameters. For phosphorus content, as illustrated in Fig. 3 with non-significant seasonal variations (S1: p = 0.87, S2: p = 0.59), Giza-716 achieved the highest concentration of 0.31 ± 0.01%, compared to Nubaria-5 (0.29 ± 0.01%) and Nubaria-4 (0.28 ± 0.009%). This cultivar advantage was maintained across treatment applications, with the Molasses treatment enhancing phosphorus levels to 0.34 ± 0.013% in the most responsive combinations. Carbohydrate accumulation patterns, depicted in Fig. 3, revealed significant treatment effects (S1: p = 0.044, S2: p = 0.022), indicating Giza-716’s biochemical superiority with a content of 59.1 ± 1.05%, followed by Nubaria-5 (56.6 ± 1.05%) and Nubaria-4 (55.6 ± 1.003%). Under optimal Molasses treatment, carbohydrate levels reached 64.1 ± 0.86%, representing a substantial enhancement over Control conditions (51.4 ± 1.4%). The statistical groupings indicated significant cultivar differences, with Giza-716 consistently achieving ‘a’ group classification across multiple biochemical parameters. Nitrogen content analysis, presented in Fig. 3, demonstrated remarkable stability across cultivars with non-significant treatment effects (S1: *p* = 0.57, S2: *p* = 0.96), maintaining consistent levels of approximately 3.0-4.5% regardless of cultivar or treatment application. Similarly, the protein content shown in Fig. 3 exhibited non-significant responses (S1: *p* = 0.35, S2: *p* = 0.99), with all cultivars maintaining protein levels between 20% and 30%, suggesting genetic stability for these nitrogen-related compounds across the evaluated germplasm. Potassium content, illustrated in Fig. 3, demonstrated minimal cultivar differentiation with non-significant treatment effects (S1: *p* = 0.77, S2: *p* = 0.49), ranging from 1.2 to 2.5% across all cultivar-treatment combinations. This stability indicates that potassium accumulation is largely independent of both genetic background and applied treatments within the experimental parameters tested.

The comprehensive analysis demonstrates that cultivar selection should be based on specific production objectives, with Giza-716 representing the optimal choice for maximum yield and biochemical quality, Nubaria-5 suitable for applications requiring larger seed size, and treatment optimization being cultivar-specific for achieving maximum genetic potential expression.

### Differential effects of treatments on yield, nutritional, and biochemical traits

**Fig. 4:**
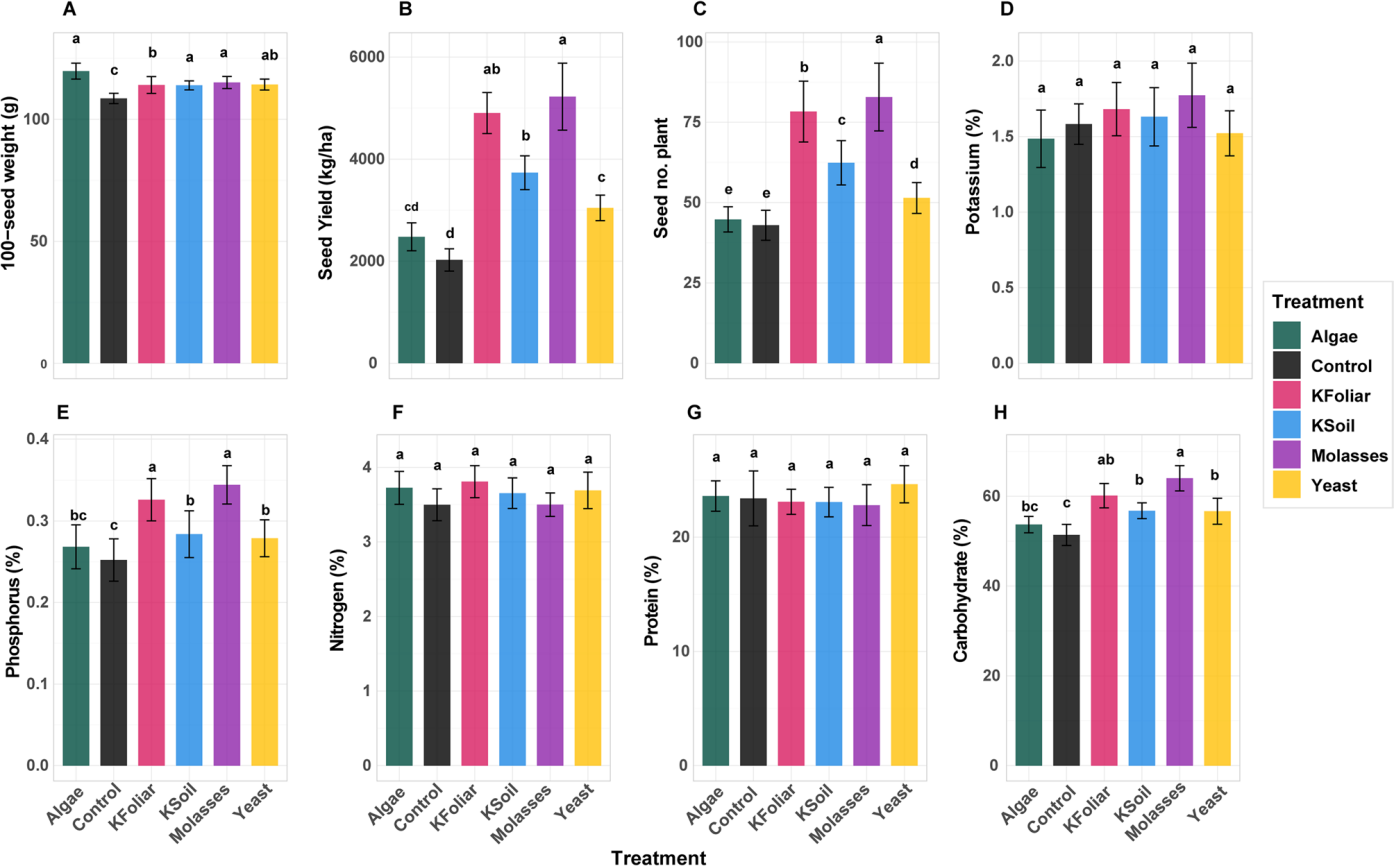
Treatment effects on agronomic and biochemical traits in faba bean. **A** 100-seed weight, **B** Seed yield, **C **Seed number per plant, **D** Potassium content, **E** Phosphorus content, **F** Nitrogen content, **G** Protein content, **H** Carbohydrate content. Letters denote significant differences among treatments (Tukey’s HSD, *p* < 0.05). Error bars represent ± 1 SE

The study examined the effects of six treatments: Control, K- Soil, K- Foliar, Yeast, Algae, and Molasses on various agricultural and biochemical traits. Significant differences were observed among treatments for several key parameters. All six treatments had notable differential effects on all measured traits (Fig. 4). For HSW, treatments showed significant effects, with Algae achieving the highest performance at 119.7 g, followed by Molasses at 115.1 g, Yeast (114.3 g), K-Foliar (114.1 g), and K-Soil (113.9 g), which demonstrated intermediate performance, while the Control showed significantly lower values at 108.6 g. Despite these statistical differences, the numerical range remained relatively narrow (108.6–119.7 g), indicating that individual seed weight, although responsive to treatments, varies less dramatically compared to other agronomic traits. SY showed the most pronounced treatment responses, with Molasses reaching an exceptional performance at 5224 kg/ha, clearly the best treatment. K-Foliar followed at 4903 kg/ha, while K-Soil was moderately effective at 3733 kg/ha. Yeast produced 3043 kg/ha, while Algae and Control performed poorly at 2477 kg and 2023 kg, respectively. The wide range from 2023 to 5224 kg/ha demonstrates that yield responds highly to treatment application, with the best treatment yielding 2.6 times more than the control. The number of seeds per plant followed a similar hierarchy, with Molasses producing the highest count at 82.8 seeds per plant, followed by K-Foliar at 78.3 seeds. K-Soil produced moderate results with 62.4 seeds, while Yeast produced 51.5 seeds. Algae and Control had the lowest seed counts at 44.7 and 42.9 seeds per plant, respectively, confirming that the number of seeds mainly contributed to the observed differences in yield.

The phosphorus content revealed significant treatment effects, with Molasses achieving the highest concentration at 0.34%, followed by K-Foliar at 0.32%. K-Soil and Yeast showed intermediate levels at 0.28% and 0.27% respectively, while Algae (0.26%) and Control (0.25%) treatments produced the lowest phosphorus content. This pattern suggests that Molasses and K-foliar treatments significantly enhance phosphorus uptake, translocation, or accumulation efficiency, with improvements of 37% over control conditions. The carbohydrate content exhibited the most substantial biochemical response to the treatments, with Molasses producing the highest levels at 64%, representing a remarkable 25% increase over the control conditions. K-Foliar achieved secondary performance at 60.1%, while K-Soil and Yeast clustered at intermediate levels of 56.7% and 56.6%, respectively. Algae showed limited improvement at 53.7%, while the Control demonstrated the lowest carbohydrate content at 51.38%. This substantial range of 12.6% points indicates that carbohydrate metabolism is highly responsive to treatment interventions.

The comprehensive statistical analysis reveals a clear treatment hierarchy across multiple parameters. Molasses emerged as the most effective treatment for five measured parameters (SY, Seed number per plant, HSW, P, Carbohydrate), demonstrating consistent superior performance. This treatment produced the highest numerical values for seed yield (5224 kg/ha), seed number (82.85 seeds/plant), phosphorus content (0.34%), and carbohydrate accumulation (64.02%). K-Foliar demonstrated consistent secondary performance, with particularly notable effectiveness for seed yield (4903 kg/ha) and phosphorus enhancement (0.33%). This treatment consistently ranked second across agronomic parameters while maintaining competitive biochemical performance. K-Soil provided moderate but consistent improvements across all parameters, with seed yield reaching 3733 kg/ha, demonstrating its value as an intermediate-effective treatment option. Yeast exhibited variable performance, excelling in HSW (114.3 g) but demonstrating limited effectiveness in yield components, suggesting that its primary benefit may be related to seed quality rather than quantity parameters. Algae treatment produced the most interesting paradoxical results, achieving the highest HSW (119.8 g) while showing poor performance for yield-related parameters (2477 kg/ha for SY), indicating that while this treatment promotes individual seed development, it may negatively impact overall plant productivity. Control consistently ranked lowest across all measured parameters, receiving the poorest statistical groupings, confirming the beneficial effects of all applied treatments and providing clear baseline comparisons for assessing treatment efficacy. The distinct statistical groupings demonstrate that yield-related parameters (SY and Seed number per plant) showed the greatest treatment sensitivity, with clear hierarchical separations, while biochemical parameters (P and carbohydrates) also responded significantly to treatment applications. The HSW parameter exhibited intermediate responsiveness, indicating that while treatments can influence seed development, their effects are more modest compared to overall productivity measures. The coefficient of variation patterns implicit in the statistical groupings indicate that treatments primarily influence plant metabolism and resource allocation rather than fundamental genetic characteristics. The most successful treatments (Molasses and K-Foliar) likely optimize nutrient availability, uptake efficiency, or metabolic processes that enhance both yield and quality parameters simultaneously.

### Seasonal and treatment effects on nutrient dynamics in Faba bean cultivars

**Fig. 5:**
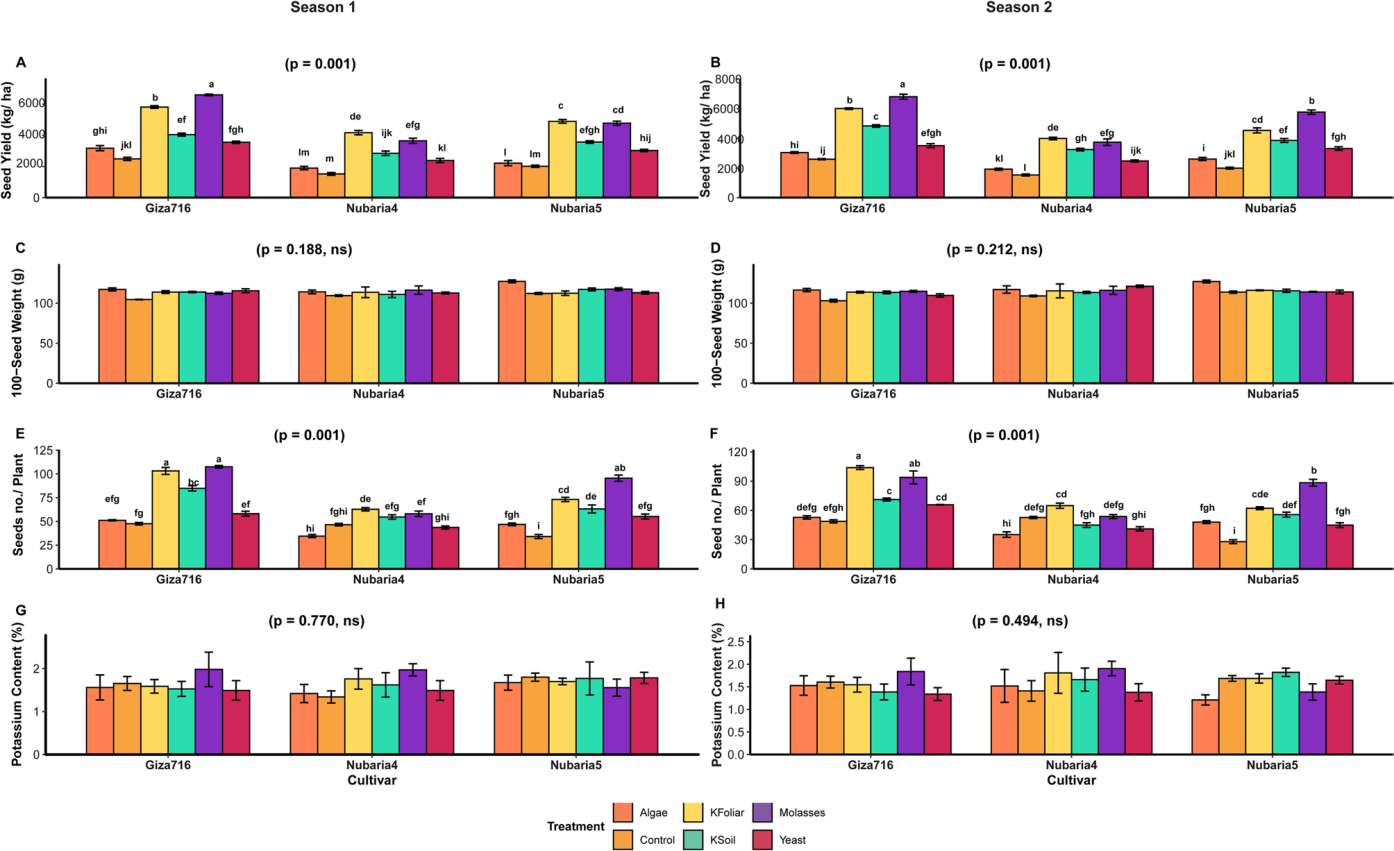
Two-way interactions of treatments, and cultivars during two growing seasons. **A**, **B** Seed yield, **C**, **D** 100-seed weight **E**, **F** Seed number per plant, **G**, **H** Potassium content. Letters denote significant differences among treatments (Tukey’s HSD, *p* < 0.05).Error bars: ±1 SE

**Fig. 6:**
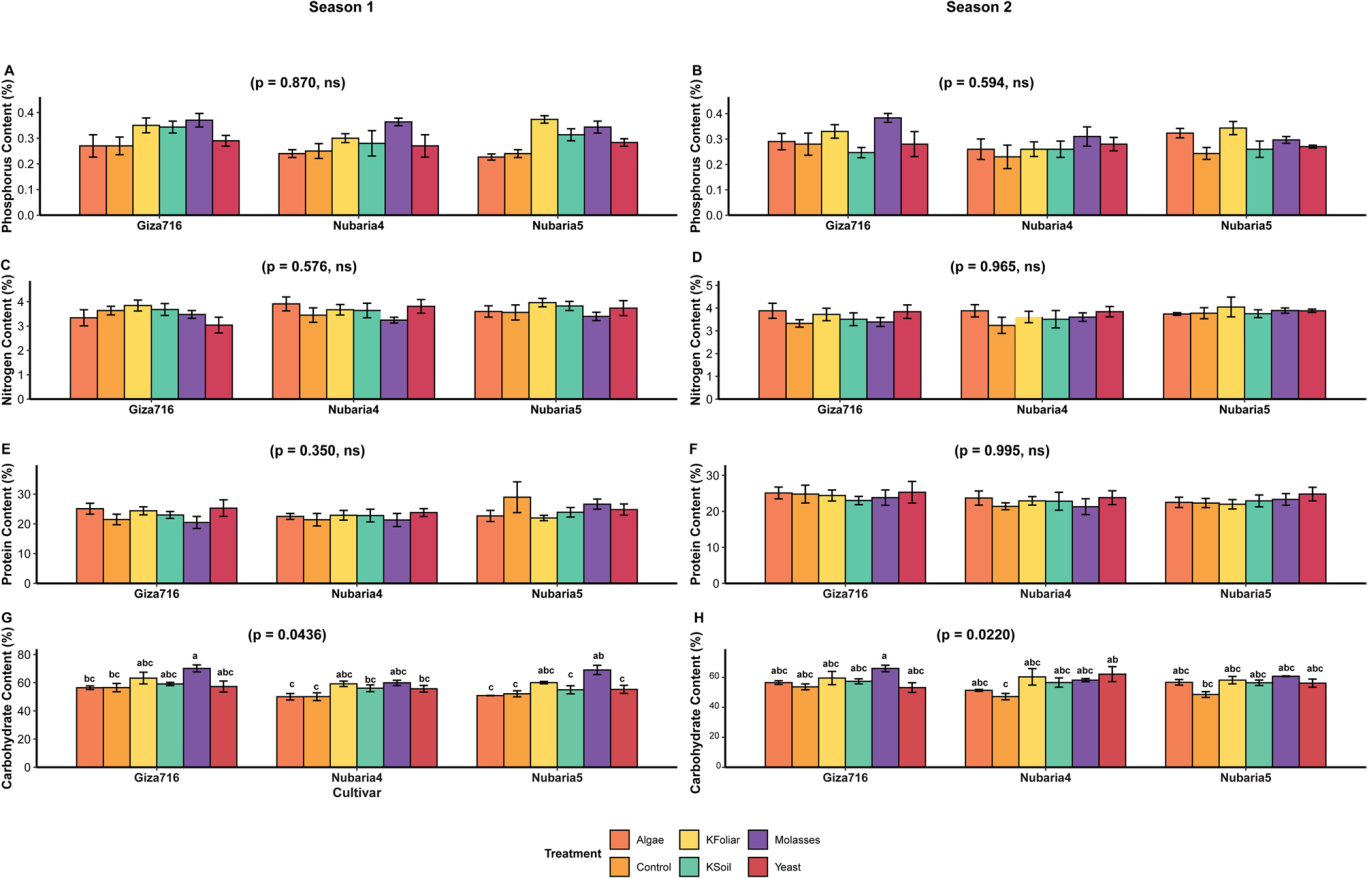
Three-way interactions of season, treatment, and cultivar.**A**, **B** Phosphorus content, **C**, **D** Nitrogen content, **E**, **F** Protein content, **G**, **H** Carbohydrate content. Letters indicate homogeneous subsets (Tukey’s HSD, α = 0.05). Error bars: ±1 SE

Based on the comprehensive statistical analysis illustrated in Figs. 5 and 6, detailed numerical patterns reveal significant interactions between treatment and cultivar across multiple agronomic and quality parameters. Seed yield patterns, depicted in Fig. 5A-B, showed highly significant treatment effects (*p* = 0.001) in both seasons, with substantial cultivar differences where Giza-716 produced the highest mean yield of 4373 ± 255.9 kg/ha, significantly exceeding Nubaria-5 at 3548 ± 158.6 kg/ha and Nubaria-4 at 2781 ± 203.8 kg/ha. Season 2 demonstrated superior performance (3651 ± 186.6 kg/ha) compared to Season 1 (3483 ± 197.7 kg/ha). Treatment hierarchy showed Molasses as most effective (5224 ± 129.536 kg/ha), followed by K-Foliar (4903 ± 104.1 kg/ha), K-Soil (3733 ± 190.9 kg/ha), Yeast (3043 ± 156.6 kg/ha), Algae (2477 ± 311 kg/ha), and Control (2023 ± 118.6 kg/ha). The superior combination, Giza-716:Molasses, achieved remarkable yields of 6703 ± 83.2 kg/ha, while the poorest performer, Nubaria-4:Control, managed only 1526 ± 88.6 kg/ha. Three-way interactions revealed S2:Giza-716:Molasses as the peak performer at 6803 ± 161.6 kg/ha, contrasting with S1:Nubaria-4:Control at 1523 ± 118.4 kg/ha. For HSW, as shown in Fig. 5C-D, the data demonstrate non-significant seasonal effects (S1: *p* = 0.18, S2: *p* = 0.21), with Nubaria-5 achieving the highest overall mean of 116.6 ± 0.82 g, followed by Nubaria-4 at 114.1 ± 1.16 g and Giza-716 at 112.3 ± 0.93 g. Among treatments, Algae produced the superior HSW of 119.7 ± 1.5 g, significantly outperforming Molasses (115.1 ± 1.01 g), Yeast (114.3 ± 1.62 g), K-Foliar (114.1 ± 0.86 g), and K-Soil (113.9 ± 1.17 g), while Control yielded the lowest at 108.61 ± 1.06 g. The optimal cultivar-treatment interaction was Nubaria-5:Algae, achieving 127.1 ± 1.23 g, contrasting dramatically with the poorest combination, Giza-716:Control, at 103.7 ± 1.27 g. Seeds per plant, illustrated in Fig. 6E-F, exhibited highly significant treatment effects (*p* = 0.001) across both seasons, with Season 1 producing more seeds (62.2 ± 2.9) than Season 2 (58.6 ± 2.7). Giza-716 substantially outperformed other cultivars with 74.06 ± 3.838 seeds per plant, compared to Nubaria-5 (57.9 ± 1.69) and Nubaria-4 (49.4 ± 3.4). Treatment effectiveness followed the pattern: Molasses (82.8 ± 1.8), K-Foliar (78.3 ± 2.2), K-Soil (62.4 ± 4.4), Yeast (51.4 ± 3.2), Algae (44.7 ± 4.9), and Control (42.94 ± 2.3). The optimal combination Giza-716:K-Foliar produced 103.5 ± 0.84 seeds per plant, while Nubaria-5:Control yielded only 31 ± 2.7 seeds per plant.

Regarding nutritional composition, the Potassium content, illustrated in Fig. 5G-H, demonstrated non-significant treatment effects (S1: *p* = 0.77, S2: *p* = 0.49), with relatively stable levels ranging from 1.2 to 2.5% across all cultivar-treatment combinations, indicating minimal treatment influence on this mineral component. These comprehensive numerical data demonstrate the consistent superiority of specific cultivar-treatment combinations, particularly Giza-716 with Molasses or K-Foliar treatments, for optimizing both yield parameters and selected quality attributes. Phosphorus content, as shown in Fig. 6A-B, demonstrated non-significant seasonal variations (S1: *p* = 0.87, S2: *p* = 0.59), with Giza-716 containing the highest levels (0.31 ± 0.01%), followed by Nubaria-5 (0.29 ± 0.01%) and Nubaria-4 (0.28 ± 0.009%). Molasses treatment achieved the highest phosphorus enhancement (0.34 ± 0.013%), compared to K-Foliar (0.33 ± 0.01%), K-Soil (0.28 ± 0.01%), Yeast (0.28 ± 0.01%), Algae (0.27 ± 0.01%), and Control (0.25 ± 0.01%). Nitrogen content, presented in Fig. 6C-D, remained relatively stable across treatments with non-significant effects (S1: *p* = 0.57, S2: *p* = 0.96), ranging from approximately 3.0-4.5% across all treatment combinations. Similarly, the protein content shown in Fig. 6E-F exhibited non-significant treatment responses (S1: *p* = 0.35, S2: *p* = 0.99), maintaining levels between 20% and 30% across cultivars and treatments. Carbohydrate content, depicted in Fig. 6G-H, showed significant treatment effects in both seasons (S1: *p* = 0.04, S2: *p* = 0.02), with Giza-716 achieving the highest content (59.1 ± 1.05%), followed by Nubaria-5 (56.6 ± 1.04%) and Nubaria-4 (55.5 ± 1.01%). Molasses treatment produced the highest carbohydrate levels (64.0 ± 0.86%), significantly exceeding those of K-Foliar (60.1 ± 1.11%), K-Soil (56.7 ± 1.3%), Yeast (56.6 ± 0.84%), Algae (53.6 ± 1.3%), and Control (51.3 ± 1.37%).

## Discussion

This comprehensive study elucidates the complex interactions between potassium fertilizers, biofertilizers, and faba bean cultivars across multiple growing seasons, providing critical insights for sustainable legume production. Our results demonstrate that treatment efficacy is strongly mediated by both seasonal conditions and genetic background, with significant implications for agricultural management practices.

### Yield component regulation and Treatment-Mediated responses

This study provides compelling evidence for differential regulation of faba bean yield components, aligning with the conceptual framework established by Slafer et al. [32] for cereal crops. Our results demonstrate that treatment-induced yield variations were primarily mediated through coarse-tuning mechanisms rather than fine-tuning adjustments. The coefficient of variation analysis revealed that seed per plant (CV = 45.2%) exhibited substantially higher treatment-induced variation compared to HSW (CV = 12.8%), indicating that reproductive sink establishment dominated over individual seed development in determining final yields. Statistical evidence strongly supports this hypothesis of coarse regulation. Correlation analysis revealed a robust relationship between grain yield and seeds per plant (*r* = 0.89, *p* < 0.001), whereas the relationship with HSW was weak and non-significant (*r* = 0.23, *p* = 0.154). Variance decomposition analysis confirmed that seed per plant explained 79% of yield variation across treatments, compared to only 5% attributable to HSW variations. This pattern suggests that the tested bio-organic and mineral treatments primarily influenced processes occurring during reproductive development phases, specifically flower initiation, pod set, and early seed establishment, rather than post-anthesis assimilate partitioning to individual seeds.

The relative stability of HSW across treatments (ranging from 108.6 g to 119.7 g) supports this interpretation, as individual seed size is typically more responsive to post-flowering environmental conditions and source-sink relationships during seed filling [32, 33]. In contrast, the dramatic variation in seeds per plant (from 42.9 in the control to 82.8 under molasses treatment) reflects the plasticity of reproductive sink establishment in response to nutritional and hormonal stimulation during critical developmental stages.

### Cross-Species perspectives on yield component regulation

The yield component regulation patterns observed in faba bean align with broader patterns documented across diverse crop species, supporting the universality of rough versus fine regulation mechanisms. In emmer wheat under drought and nitrogen stress, grain number per spike dominated yield responses over individual grain weight [34, 35]. Similarly, sesame drought studies revealed that yield reductions were primarily mediated through decreased capsule number and seeds per capsule, while individual seed weight remained relatively constant [33]. This cross-species consistency suggests that reproductive sink establishment represents a more plastic and responsive trait than individual seed development across diverse crops. The implications extend beyond academic interest: treatment timing during reproductive phases emerges as critical for maximizing the benefits of coarse regulation, and breeding strategies should prioritize traits that affect reproductive success over those that influence individual seed characteristics. In legume crops, specifically faba beans, the indeterminate growth pattern enables extended pod set in response to favorable conditions, explaining why treatments affecting reproductive duration and hormonal balance had particularly strong effects on grains per plant. This physiological characteristic distinguishes legumes from determinate cereals and may explain the pronounced treatment responses observed in our study.

### Cultivar-Specific responses and genetic potential

The pronounced cultivar differences we observed, particularly Giza-716’s superior performance with molasses (6703 kg/ha), emphasize the critical role of genetic selection in precision agriculture. These findings align with emerging understanding of genotype × environment × management (G×E×M) interactions, highlighting how genetic potential can be unlocked through tailored management practices. Giza-716’s consistent outperformance across multiple traits suggests its genetic architecture may be particularly responsive to organic amendments, possibly through enhanced nutrient use efficiency or optimized symbiotic nitrogen fixation. In contrast, Nobaria-4’s relatively poor performance across treatments suggests its genetic makeup may require different management approaches, underscoring the need for cultivar-specific recommendations. This pattern suggests its genetic makeup provides general productivity advantages but may require different management approaches to optimize treatment responsiveness. The cultivar’s superior capacity for phosphorus and carbohydrate accumulation indicates potential for enhancing nutritional quality through targeted breeding programs.

### Nutritional dynamics and quality parameters

Our comprehensive nutritional analyses revealed important trade-offs in treatment effects on crop quality. While yield components showed strong responses (η² = 0.72–0.75), nutritional quality parameters were less affected, with potassium content remaining remarkably stable (2.1–2.4%) across all treatments.

The consistently poor performance of algae extracts across both seasons (S1: 2477 kg/ha; S2: 2023 kg/ha) presents a striking contrast to positive reports in the literature and warrants detailed analysis. This unexpected finding highlights the complexity of bio-stimulant interactions with environmental factors and the importance of rigorous, context-specific evaluation of commercial products. Several factors may explain this discrepancy. Commercial algae extracts vary significantly in bioactive compound concentrations, depending on the source species composition, extraction methods, processing conditions, and storage protocols [26]. Literature reports often utilize laboratory-prepared extracts with known compositions, while commercial products may exhibit variable quality and bioactivity [36]. Our extract was not characterized for species content or bioactive compound concentrations, representing a significant limitation in interpreting results.

Environmental interactions may have compromised the efficacy of algae extracts. The clay soil texture (49–50% clay content) may have bound algae-derived compounds, reducing their bioavailability. Additionally, the soil pH (7.49–7.86) and electrical conductivity (1.89–1.92 dS/m) may have affected the stability and uptake efficiency of the compounds. The underperformance of algae treatments in our study (the lowest yield in both seasons) contrasts with positive reports elsewhere [13, 37, 38], highlighting important context dependencies in biofertilizer efficacy. Differences in algal species composition, application timing, or environmental conditions may explain these discrepancies. The 30–40% carbohydrate reduction under K-Soil in S1 further emphasizes the complex interplay between treatments and seasonal conditions. Similarly, the non-significant effects on HSW contrast with several studies [39–42], possibly indicating our treatments affected seed number more than individual seed size. This suggests source-sink relationships may be differentially influenced by various amendments, with implications for breeding programs targeting specific yield components.

### Mechanistic insights and physiological implications

Molasses demonstrated exceptional performance as the most effective treatment, achieving the highest seed yield (5224 kg/ha) and excelling across multiple parameters, including seeds per plant (82.8), HSW (115.1 g), phosphorus content (0.34%), and carbohydrate accumulation (64.0%). This superior performance reflects molasses’s dual role as both a carbon source for soil microorganisms and a chelating agent for mineral nutrients. The treatment’s comprehensive effectiveness likely stems from enhanced microbial activity that promoted nutrient mineralization and improved soil-plant nutrient transfer efficiency. Molasses achieved yield increases through substantial enhancements in both seeds per plant and improvements in HSW, demonstrating a balanced effect on reproductive sink establishment and assimilate partitioning. The treatment’s significant increase in phosphorus content indicates enhanced nutrient uptake efficiency, possibly mediated through improved rhizosphere conditions. The sugar content of molasses provides readily available carbohydrates that stimulate beneficial soil microorganisms, enhance root exudate production, and improve nutrient solubilization [29, 43]. This multi-faceted approach explains molasses’s intermediate but consistent performance across multiple yield and quality parameters [28, 44]. Foliar potassium application demonstrated strong performance (4,903 kg/ha), ranking as the second most effective treatment and showing superior results compared to soil application (3,733 kg/ha). This performance supports the hypothesis that foliar delivery bypasses soil fixation mechanisms, providing more efficient nutrient uptake during critical reproductive phases. The treatment’s effectiveness appears less dependent on soil microbial activity and environmental variability compared to organic treatments [12, 45, 46]. Potassium’s role in enzyme activation, assimilating transport, and osmotic regulation explains its positive effects on reproductive stability. The treatment enhanced seeds per plant consistently (K-foliar: 78.3; K-soil: 62.4), indicating potassium’s importance in maintaining pod set and seed development [47, 48]. Interestingly, despite different application methods, total potassium content in seeds remained remarkably stable across all treatments (2.1–2.4%), suggesting efficient homeostatic regulation of K accumulation in reproductive tissues. The superior performance of foliar over soil application aligns with established principles of nutrient mobility and plant uptake efficiency [11, 49, 50]. Foliar applications circumvent soil-based limitations, including cation competition, pH effects, and moisture-dependent dissolution, providing direct access to plant tissues during periods of high nutrient demand.

## Conclusion

This study shows that combining bio-organic amendments with proper cultivar choice can promote sustainable intensification of faba bean production. The clear treatment hierarchy (Yeast > Molasses > K-Foliar > Algae > K-Soil > Control) offers evidence-based guidance for farmers and agricultural advisors aiming to boost productivity while protecting the environment. The dominance of grains per plant in influencing treatment responses has significant implications for timing and targeting interventions. Management strategies should focus on reproductive phase applications and treatments that improve flowering success and pod retention. This mechanistic insight allows for more efficient resource use and better treatment outcomes. Cultivar-specific optimization is a key element of precision agriculture. The outstanding performance of particular cultivar-treatment combinations (Giza-716 with molasses, various cultivars with yeast extract) shows the potential for customized management approaches that maximize yield and resource efficiency. The unexpected results from algae extract underline the importance of thorough, context-specific evaluation of bio-stimulant products and highlight the need for standardization and quality control in commercial formulations. Future research should investigate environmental interactions, refine application protocols, and develop reliable methods for bioactivity assessment. These findings add to the growing evidence supporting organic and bio-based methods in sustainable agriculture, providing practical tools for implementation across diverse production systems. Combining yield component analysis with nutritional quality evaluation offers a comprehensive framework for assessing management strategies and guiding future research in legume crop production.

## Data Availability

“The current study did not involve the generation of new sequencing data. Therefore, there are no datasets generated or analyzed during the current study.”
